# Fall detection and pre-impact prediction technologies in older adults: a scoping review of translational maturity and public health integration

**DOI:** 10.3389/fpubh.2026.1737644

**Published:** 2026-03-25

**Authors:** Li Chen, Wu Yao

**Affiliations:** 1 Department of Physical Education Teaching, Shanghai Sanda University, Shanghai, China; 2 Department of Physical Education, Shanghai Jiao Tong University, Shanghai, China

**Keywords:** fall detection, older adults, pre-impact prediction, translational maturity, wearable sensors

## Abstract

**Objective:**

To map the current landscape of wearable and sensor-based fall detection and pre-impact prediction technologies relevant to older adults and to evaluate their translational maturity within public health contexts.

**Methods:**

A scoping review was conducted following PRISMA-ScR guidelines. Four electronic databases (PubMed, Web of Science, Scopus, and IEEE Xplore) were systematically searched for studies published between January 2005 and September 2025. Eligible studies reported the development or validation of fall detection or pre-impact prediction systems incorporating wearable, vision-based, environmental, or multimodal sensing modalities. In total, 243 studies were included in the overall synthesis, with a predefined subgroup of 21 studies involving real-world or mixed real-world validation in older adult populations (≥65 years).

**Results:**

Across the 243 included studies, wearable inertial measurement unit (IMU)-based systems constituted the dominant technological stream, and post-fall detection remained the most frequently investigated functional objective. However, more than half of studies relied primarily on laboratory-based simulated fall protocols. Within the real-world validated older adult subgroup (*n* = 21), 71.4% focused on post-fall detection, 19.0% investigated pre-impact prediction, and 9.5% addressed fall risk modeling. While technical performance metrics such as sensitivity and specificity were frequently reported under controlled conditions, evidence regarding long-term adherence, workflow integration, and health economic impact was limited. A maturity gradient emerged across modalities, with wearable detection systems demonstrating stronger ecological grounding than predictive, multimodal, and ecosystem-level approaches.

**Conclusion:**

Although technological innovation in fall-related sensing systems has expanded rapidly, translational maturity remains uneven. Bridging the gap between algorithmic performance and scalable public health implementation will require robust real-world validation, longitudinal adherence evaluation, implementation science frameworks, and economic assessment. Advancing along a continuum from reactive detection toward predictive and personalized prevention represents a critical pathway for supporting safe and independent aging.

## 1 Introduction

### 1.1 Global aging and the public health burden of falls

Population aging represents one of the most profound demographic transformations of the twenty-first century. According to global demographic projections, the proportion of individuals aged 60 years and older is rapidly increasing across both high-income and low- and middle-income countries (1). This demographic shift is accompanied by a parallel rise in age-related morbidity, functional decline, and health system demand. Among the many health challenges facing aging societies, falls remain one of the most pressing and preventable threats to older adults’ well-being (2, 3).

Falls are a leading cause of injury-related hospitalization, disability, and mortality in older populations. Beyond acute trauma—such as fractures, head injuries, and soft tissue damage—falls often trigger a cascade of secondary consequences (4), including fear of falling, reduced mobility, physical deconditioning, and social withdrawal (5). These downstream effects substantially increase long-term healthcare costs and accelerate transitions from independent living to assisted care or institutionalization (6).

From a public health perspective, falls are not isolated biomechanical events but complex outcomes of interactions between physiological vulnerability, environmental hazards, behavioral patterns, and social determinants of health (7). Therefore, fall prevention must be framed within a broader aging and public health context rather than treated solely as an injury detection problem.

### 1.2 Beyond injury: quality of life, functional autonomy, and social participation

While fall-related fractures and traumatic injuries are highly visible outcomes, the broader impact of falls on quality of life and functional autonomy is equally significant (2). Recurrent falls and fear of falling frequently lead to activity restriction, decreased participation in daily living tasks, and reduced engagement in social and physical activities (2). These behavioral adaptations may contribute to progressive muscle weakness, impaired balance, and diminished cardiovascular fitness, thereby increasing future fall risk in a self-reinforcing cycle.

Healthy aging frameworks emphasize the maintenance of intrinsic capacity, functional ability, and meaningful participation in society. Muscle strength, mobility, and physical activity participation are strongly associated with preserved independence in activities of daily living (8, 9). Conversely, social isolation and reduced engagement may negatively influence both psychological well-being and physical resilience. Thus, fall prevention technologies should not merely aim to detect impact events but should support broader goals of maintaining independence, dignity, and social participation among older adults.

In this context, intelligent monitoring and predictive systems may serve as enabling tools within a holistic healthy aging strategy. However, technological solutions must align with older adults’ preferences, usability needs, and cultural contexts to ensure acceptance and sustained utilization (10, 11).

### 1.3 Evolution of fall detection and prediction technologies

Over the past two decades, technological innovation has transformed the landscape of fall monitoring systems. Early-generation devices primarily relied on threshold-based accelerometer algorithms to detect high-impact events (12, 13). These systems were largely reactive, activating alarms after a fall had already occurred.

Subsequent developments introduced machine learning classifiers capable of distinguishing falls from activities of daily living using multi-axis inertial data (14, 15). Improvements in computational power and miniaturization facilitated wearable integration and real-time processing.

More recently, research attention has expanded toward pre-impact fall prediction, multimodal sensor fusion, and context-aware monitoring. These systems attempt to identify instability patterns during the descent phase or to integrate complementary signals—such as barometric pressure, environmental context, or physiological indicators—to improve robustness (16, 17). Parallel research streams have explored vision-based and depth-sensor technologies capable of analyzing posture and gait patterns without requiring wearable devices (18, 19).

Emerging conceptual frameworks, including edge intelligence, digital twin-inspired modeling, and distributed learning architectures, propose anticipatory and adaptive fall prevention ecosystems. While promising, many of these approaches remain in early validation phases and require careful evaluation regarding feasibility, scalability, and real-world effectiveness (20).

### 1.4 Persistent challenges in translation

Despite rapid technological evolution, several persistent barriers limit large-scale implementation. A considerable proportion of studies rely on simulated falls performed under laboratory conditions, frequently involving young and healthy participants (7, 21). Such datasets may not adequately represent the biomechanical complexity and heterogeneity of falls among frail older adults.

False-positive alarms remain a common challenge, particularly during dynamic but non-fall activities (14, 22). Excessive alerts can erode user trust and reduce adherence. Furthermore, wearable systems depend on sustained compliance, battery management, and device comfort—factors that are particularly relevant in aging populations (11, 23).

Vision-based systems introduce additional concerns related to privacy and continuous surveillance, raising ethical and regulatory considerations (24). Multimodal architectures, while potentially more accurate, may increase system complexity and cost, complicating scalability within public health systems.

Collectively, these issues suggest that technological innovation alone does not guarantee successful public health integration.

### 1.5 Knowledge gap and rationale for this scoping review

Although numerous studies have evaluated fall detection and prediction systems, the literature remains fragmented across engineering, rehabilitation, gerontology, and computer science domains. Comparative synthesis across sensing modalities—including wearable, vision-based, multimodal, and anticipatory systems—remains limited, and distinctions between post-fall detection and pre-impact prediction are not consistently clarified in existing reviews. Given the rapid pace of technological innovation and the diversity of methodological approaches, a comprehensive mapping of current evidence is necessary to identify research concentrations, methodological limitations, and translational gaps (25). In particular, there remains insufficient clarity regarding how different sensing modalities compare in terms of validation context and performance reporting, the extent to which predictive systems have undergone rigorous external validation in older adult populations (8), the recurring technical and operational bottlenecks that hinder real-world deployment, and the degree to which emerging technological advances align with broader aging and public health objectives (21). Addressing these gaps is essential for advancing fall-related technologies from experimental prototypes toward scalable, sustainable, and ethically grounded public health solutions.

### 1.6 Research question and objectives

This scoping review seeks to answer the following research question: What is the current state of evidence on wearable and sensor-based fall detection and pre-impact prediction systems relevant to older adults, and what technical, ethical, and translational challenges constrain their large-scale public health implementation?

The objectives of this review are threefold: To map major technological streams, including wearable inertial systems, multimodal sensor fusion architectures, vision- and depth-based approaches, and anticipatory prediction models. To summarize reported validation contexts and performance characteristics. To identify cross-cutting barriers and opportunities for advancing predictive, preventive, and personalized fall prevention within aging and public health frameworks.

## 2 Methods

### 2.1 Study design and methodological framework

This study was conducted as a scoping review to systematically map the breadth, characteristics, and methodological trends of fall detection and pre-impact prediction technologies relevant to older adults within public health contexts. A scoping review design was selected due to the considerable heterogeneity in sensor modalities, algorithmic strategies, validation protocols, outcome measures, and study populations across the field. Unlike systematic reviews that aim to generate pooled effect estimates through meta-analysis, scoping reviews are particularly suited to emerging and multidisciplinary domains in which conceptual clarification, evidence mapping, and identification of translational gaps are primary objectives. The review followed established methodological guidance for scoping reviews and adhered to the PRISMA-ScR (Preferred Reporting Items for Systematic Reviews and Meta-Analyses extension for Scoping Reviews) reporting framework to enhance transparency and reproducibility. The research questions, eligibility criteria, data-charting domains, and synthesis strategy were defined prior to full-text screening. The review protocol was not prospectively registered.

### 2.2 Data sources and search strategy

A comprehensive literature search was conducted across four major electronic databases—PubMed, Web of Science, Scopus, and IEEE Xplore—to capture interdisciplinary contributions spanning geriatrics, rehabilitation science, biomedical engineering, artificial intelligence, and computer vision. The search covered studies published between January 2005 and September 2025. The starting year was selected to reflect the emergence of wearable inertial measurement unit (IMU)-based fall detection research and subsequent technological advances in machine learning, multimodal sensing, and edge computing. The final database search was conducted on 30 September 2025.

Search terms combined controlled vocabulary and free-text keywords across three conceptual domains: fall-related terminology (including “fall detection,” “fall prediction,” “pre-impact,” “fall risk,” and “near fall”), technological modalities (including “wearable,” “IMU,” “accelerometer,” “gyroscope,” “sensor fusion,” “multimodal,” “computer vision,” “depth sensor,” “Kinect,” “edge computing,” “digital twin,” “federated learning,” and “machine learning”), and aging population descriptors (including “older adults,” “elderly,” “aging,” and “geriatric”). Boolean operators were used to combine search terms within and across domains. Database-specific search strings are provided in the Supplementary Material to ensure reproducibility.

### 2.3 Eligibility criteria

Studies were eligible for inclusion if they reported the development, validation, or evaluation of a fall detection or pre-impact fall prediction system employing wearable, environmental, vision-based, or multimodal sensing modalities and provided at least one quantitative performance metric, such as sensitivity, specificity, F1-score, accuracy, area under the curve (AUC), false alarm rate, or prediction lead time. To maintain relevance to aging and public health contexts, studies were required to include participants aged 60 years or older or explicitly target older adult care settings, such as community-dwelling older adults, assisted living environments, or long-term care facilities. Only peer-reviewed full-text articles published in English were considered.

Studies involving simulated fall protocols were included if they involved older adult participants or were explicitly designed for geriatric applications; however, simulated and real-world validation contexts were categorized separately during synthesis. Studies were excluded if they were conference abstracts lacking sufficient methodological detail, focused exclusively on pediatric or non-aging populations, failed to report empirical validation results, or addressed fall risk solely through questionnaire-based or clinical scale assessments without incorporating technological sensing components. No restrictions were imposed regarding geographic region or care setting.

### 2.4 Study selection process

**Figure 1:**
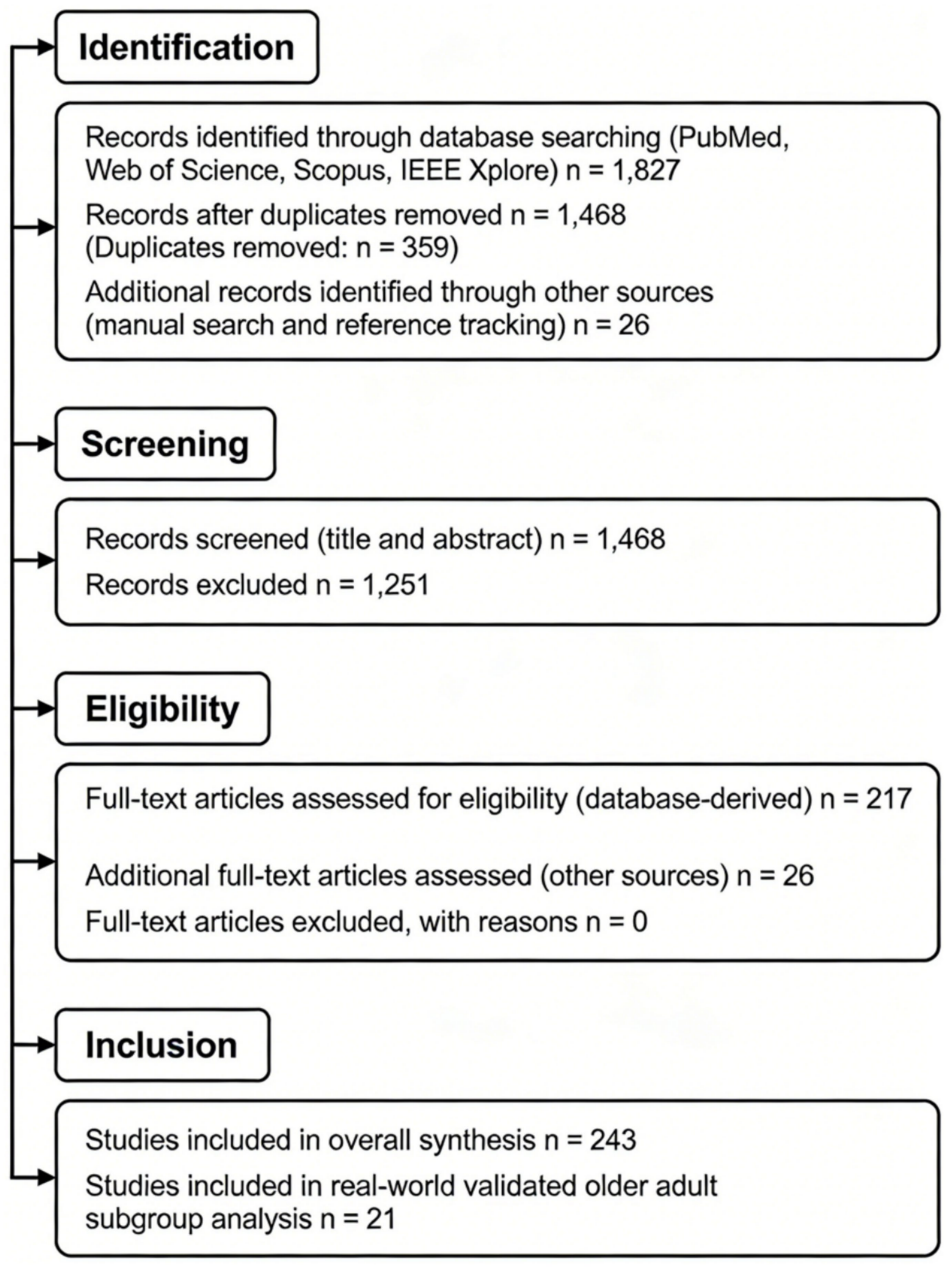
PRISMA flow diagram illustrating the study selection process.

All retrieved records were imported into reference management software, and duplicates were removed prior to screening. Two reviewers independently screened titles and abstracts against predefined inclusion criteria. Articles deemed potentially eligible underwent full-text assessment. Disagreements during screening and eligibility assessment were resolved through discussion and consensus, with consultation of a third reviewer if necessary. A total of 1,827 records were identified through systematic searches of four electronic databases (PubMed, Web of Science, Scopus, and IEEE Xplore). After removal of 359 duplicate records, 1,468 unique articles remained for title and abstract screening. Of these, 1,251 records were excluded based on irrelevance to fall detection or prediction technologies, absence of technological components, non-aging populations, or lack of empirical validation data. A total of 217 full-text articles identified from database searches were assessed for eligibility. An additional 26 studies were identified through manual searches and backward reference tracking. Following full-text evaluation and application of predefined inclusion and exclusion criteria, a total of 243 studies were included in the overall synthesis. Among these 243 included studies, 21 met additional predefined criteria for subgroup analysis, specifically involving real-world or mixed real-world validation in older adult populations aged 65 years or above. The complete study selection process is illustrated in Figure 1.

### 2.5 Data extraction and charting

A standardized data-charting form was developed prior to full extraction to ensure consistency. For each included study, the following information was extracted: authorship and publication year; country or region; study design and validation setting (laboratory-based versus real-world); sample characteristics including age range, health status, and sample size; primary sensing modality (such as IMU, vision-based systems, environmental sensors, or multimodal configurations); algorithmic approach (including threshold-based methods, supervised machine learning, deep learning, or hybrid strategies); reported performance metrics; prediction lead time for pre-impact systems; and key strengths and limitations identified by the authors. Data extraction was conducted independently by two reviewers and cross-checked to ensure accuracy and completeness.

### 2.6 Study characteristics and descriptive analysis

Included studies were categorized according to primary sensing modality and functional objective, distinguishing between post-fall detection systems and pre-impact prediction systems. Validation context was further classified as laboratory-based, including simulated fall experiments, or real-world deployment in unsupervised community or institutional environments. Descriptive statistics were used to characterize the distribution of sensing modalities, the proportion of single-modality versus multimodal systems, the relative frequency of laboratory versus real-world validation contexts, geographic distribution, sample size ranges, and the types of performance metrics reported. Given the heterogeneity in study designs, sensor configurations, validation procedures, and outcome definitions, quantitative meta-analysis was not performed.

### 2.7 Data synthesis approach

A narrative thematic synthesis approach was employed to organize the included studies into coherent technological streams. Themes were derived inductively based on sensor architecture, functional capability (detection versus prediction), system integration complexity, and validation maturity. Comparative analysis across categories was conducted to identify recurring cross-cutting challenges, including ecological validity limitations, false-positive burden, algorithm interpretability, long-term adherence constraints, privacy concerns, and scalability within public health systems. Because scoping reviews are designed to map and characterize existing evidence rather than to formally evaluate methodological quality, risk-of-bias assessment was not undertaken. Nevertheless, limitations reported by original studies were systematically summarized to contextualize performance claims and to inform discussion of translational readiness.

## 3 Results

### 3.1 Study selection and overall characteristics

A total of 243 studies met the inclusion criteria and were included in the overall synthesis (Figure 1). The included studies were published between 2005 and 2025, with a marked increase after 2015, coinciding with the widespread adoption of wearable IMU systems and machine learning integration.

Across the corpus, post-fall detection represented the dominant functional objective. Wearable IMU-based systems comprised over half of all studies, followed by vision-based approaches (~10%) and multimodal or environmental systems (~15–20%). More than half of studies relied on laboratory-based simulated protocols, whereas fewer than one-third reported real-world validation in older adult settings.

To assess translational maturity, a subgroup analysis was conducted on 21 studies involving older adults (≥65 years) with real-world or mixed validation designs. Within this subgroup, 71.4% focused on post-fall detection, 19.0% on pre-impact prediction, and 9.5% on risk modeling. Wearable IMU systems accounted for 61.9% of studies, while vision-based and multimodal approaches were less represented. Although ecological validation was more prominent in this subgroup, several studies still relied on limited-duration or semi-controlled environments.

Overall, the broader corpus reflects rapid technological expansion, whereas the real-world validated subgroup highlights uneven translational maturity. This contrast forms the basis for the maturity gradient analysis presented in subsequent sections (Table 1).

**Table 1:** Characteristics of real-world validated older adult studies (*n* = 21).

Author (year)	Setting	Sample size	Age (years)	Sensor modality	Functional objective	Validation context	Key performance metrics
Klenk et al. (2016) (41)	Multicenter real-world repository (FARSEEING)	>200 participants	≥65	Waist-worn IMU	Post-fall detection	Real-world	Sensitivity, specificity
Bourke et al. (2016) (42)	Long-term care facilities	>40 participants	≥65	Lumbar IMU	Post-fall detection	Real-world	Sensitivity, AUC
Bagalà et al. (2012) (14)	Real-world fall database	29 fall events	≥65	Accelerometer	Post-fall detection	Real-world	Sensitivity, specificity
Saleh et al. (2021) (43)	Long-term care institution	>70 participants	≥70	Wearable IMU	Post-fall detection	Real-world	Sensitivity, AUC
Can et al. (2024) (44)	Nursing home	>30 participants	≥75	Commercial wearable device	Post-fall detection	Real-world	Sensitivity, positive predictive value
Chaudhuri et al. (2015) (10)	Community dwelling	51 participants	≥65	Wearable device	Post-fall detection	Real-world	Sensitivity, specificity
Rantz et al. (2015) (6)	In-home monitoring	>20 households	≥70	Depth-based vision system	Post-fall detection	Real-world	Sensitivity
Stone and Skubic (2015) (18)	Homes of older adults	>10 households	≥70	Kinect-based vision system	Post-fall detection	Real-world	Sensitivity
Scheurer et al. (2019) (45)	Real-life elderly environment	25 participants	≥70	Waist-worn IMU	Post-fall detection	Real-world	Sensitivity, specificity
Feldwieser et al. (2014) (46)	Domestic home environment	>30 participants	≥65	Multimodal sensor system	Post-fall detection	Real-world	Sensitivity
Huang and Chan (2014) (47)	Home telecare	>50 participants	≥65	ZigBee-based system	Post-fall detection	Real-world	Sensitivity
Bourke et al. (2010) (22)	Continuous unsupervised monitoring	19 participants	≥65	Waist-worn IMU	Post-fall detection	Real-world	Sensitivity, false alarm rate
Lee and Carlisle (2011) (48)	Community	>40 participants	≥65	Smartphone accelerometer	Post-fall detection	Mixed (real-world + simulated)	Sensitivity
Alizadeh et al. (2021) (49)	Older-adult validation study	>30 participants	≥65	Waist-worn IMU	Post-fall detection	Mixed (real-world + simulated)	Sensitivity, specificity
Chan et al. (2023) (9)	Free-living longitudinal cohort	>500 participants	≥65	Wrist-worn sensor	Fall risk prediction	Real-world	AUC
Maiora et al. (2024) (50)	Outpatient clinic	>60 participants	≥65	TUG + Deep Learning	Fall risk prediction	Clinical validation	AUC
Yu et al. (2021) (51)	Benchmark dataset (KFall)	>40 participants	≥65	IMU	Pre-impact detection	Dataset-based evaluation	Lead time
Yu et al. (2020) (17)	Laboratory protocol	>20 participants	≥65	IMU	Pre-impact detection	Laboratory (simulated)	Sensitivity, lead time
Liu and Lockhart (2014) (16)	Laboratory	>30 participants	≥65	IMU	Pre-impact detection	Laboratory (simulated)	Lead time
Aziz et al. (2014) (27)	Laboratory	15 participants	≥65	Waist-worn IMU	Pre-impact detection	Laboratory (simulated)	Sensitivity, lead time
Bourke and Lyons (2008) (52)	Laboratory	>20 participants	≥65	Gyroscope	Post-fall detection	Laboratory (simulated)	Sensitivity

### 3.2 Wearable IMU-based fall detection systems

Wearable IMU-based systems remain the most extensively investigated modality in fall detection research. These systems typically utilize accelerometers and gyroscopes placed at the waist, chest, thigh, or wrist to capture rapid changes in acceleration and angular velocity associated with fall events.

Across laboratory-based validation studies, reported sensitivity values frequently exceeded 85–90%, with specificity ranging broadly depending on activity complexity and threshold configuration. Machine learning classifiers, including support vector machines, random forests, and neural networks, generally demonstrated improved discrimination compared with simple threshold-based approaches in controlled datasets.

However, performance variability was notable across studies. Differences in sampling frequency, sensor placement, training dataset composition, and feature engineering contributed to heterogeneity in reported metrics. Importantly, real-world validation often revealed lower specificity due to increased false-positive rates during abrupt but non-fall activities such as rapid sitting or dropping objects.

Another recurring limitation concerns adherence. Wearable systems depend on consistent device usage, correct placement, and battery management. Long-term adherence studies remain limited, and few investigations report user satisfaction or compliance rates over extended periods.

Overall, wearable IMU systems demonstrate technical maturity in controlled settings but require further real-world validation and user-centered optimization.

### 3.3 Pre-impact fall prediction systems

Pre-impact prediction systems aim to detect instability during the descent phase prior to ground contact. These systems typically analyze short temporal windows of kinematic signals to estimate fall likelihood in real time.

Reported lead times in experimental settings are generally within sub-second ranges, often between approximately 100 and 600 milliseconds depending on detection thresholds and modeling strategy. Such lead times may theoretically enable activation of protective mechanisms such as wearable airbags or automated assistive responses.

Despite promising conceptual implications, several limitations were consistently observed. Most validation datasets were generated under simulated fall conditions, often involving younger participants. Real-world falls in older adults may involve slower descent velocities, compensatory reactions, and environmental constraints not represented in controlled simulations. Consequently, generalizability remains uncertain.

Furthermore, predictive systems must balance sensitivity with false alarm minimization. Overly sensitive thresholds may generate frequent alerts during normal movements, reducing user trust and increasing alert fatigue.

Thus, while pre-impact systems represent an important conceptual advancement, their translational readiness remains limited relative to post-fall detection technologies.

### 3.4 Vision- and depth-based systems

Vision-based and depth-sensing systems form a parallel research stream in fall detection and risk assessment. These systems analyze body posture, motion trajectories, and environmental context using RGB cameras, depth sensors, or structured-light devices such as Kinect platforms.

Performance metrics reported in controlled indoor environments are frequently comparable to wearable-based systems, particularly when deep learning-based pose estimation models are employed. Vision-based systems offer the advantage of non-wearability, reducing user compliance requirements.

However, privacy considerations and environmental sensitivity remain significant challenges. Lighting variability, occlusion, furniture arrangement, and multi-person scenes can degrade detection accuracy. Moreover, continuous video monitoring raises ethical concerns regarding autonomy and dignity, particularly in private residential settings.

Most vision-based studies were conducted in assisted-living or laboratory mock-up environments. Large-scale real-world deployments remain limited, and long-term acceptance data among older adults are scarce.

### 3.5 Multimodal sensor fusion and edge computing

Multimodal systems integrate IMUs with complementary modalities such as barometric pressure sensors, electromyography, floor vibration sensors, or environmental sensors. These architectures aim to reduce false positives and enhance contextual awareness.

Compared with single-modality systems, multimodal approaches frequently report improved robustness in differentiating falls from high-dynamic daily activities. Edge computing implementations allow on-device inference, reducing latency and potential privacy risks associated with cloud transmission.

Nevertheless, increased hardware complexity may elevate cost, maintenance demands, and calibration requirements. Interoperability across heterogeneous devices remains a technical barrier. Furthermore, few studies provide cost-effectiveness analyses or large-scale community validation.

Thus, while multimodal systems may enhance classification precision, their scalability and feasibility within public health systems require further investigation.

### 3.6 Emerging intelligent ecosystems: digital twin, BCI, and distributed learning

A smaller subset of studies explores advanced ecosystem-level approaches integrating biomechanical modeling, adaptive feedback loops, and distributed learning frameworks. Digital twin-inspired models attempt to simulate individualized gait dynamics and instability thresholds. Brain–computer interface (BCI) approaches investigate neural correlates of balance control to inform anticipatory interventions. Federated learning frameworks aim to enable cross-institutional model training without centralized data pooling.

These approaches remain largely at proof-of-concept or early validation stages. Empirical data involving frail older adults are limited, and feasibility, cost, and regulatory compliance require further study. While these technologies align conceptually with predictive and personalized paradigms, their current maturity level is substantially lower than that of wearable detection systems.

### 3.7 Cross-cutting patterns and maturity gradient

When analyzed collectively, the included studies reveal a maturity gradient across technological streams. Wearable IMU-based post-fall detection systems exhibit the highest degree of technical validation, particularly in controlled laboratory environments. Pre-impact prediction systems demonstrate conceptual advancement but limited ecological validation. Vision-based systems offer non-wearable advantages yet face privacy and environmental robustness challenges. Multimodal and ecosystem-level approaches show potential for enhanced precision but remain constrained by complexity and scalability concerns.

Across all categories, the most consistent limitations include reliance on simulated fall datasets, limited external validation across diverse aging populations, insufficient reporting of long-term adherence, and absence of health economic evaluation.

These findings indicate that technological innovation in fall prevention is advancing rapidly; however, translational maturity varies substantially across modalities. Bridging this maturity gradient represents a key challenge for advancing scalable public health implementation.

## 4 Discussion

### 4.1 From reactive detection to anticipatory risk management

The evolution of fall-related technologies over the past two decades reflects a broader conceptual shift in geriatric care—from reactive injury response toward anticipatory risk management (26). Early systems were primarily designed to detect high-impact events after ground contact, focusing on rapid alert activation (12, 13). These threshold-based accelerometer systems represented an important first step in digital fall surveillance. However, their clinical value was inherently limited to post-event response.

Subsequent generations introduced machine learning–based classifiers capable of distinguishing falls from activities of daily living with greater discrimination capacity (14, 27). This transition marked a methodological maturation rather than a fundamental conceptual transformation: the objective remained event detection, albeit with improved accuracy.

More recently, research attention has expanded toward pre-impact prediction and instability monitoring. Instead of identifying the moment of impact, these systems attempt to detect biomechanical instability during the descent phase or even earlier through gait irregularities and contextual signals (16, 17, 28). This transition reflects a deeper reorientation—from documenting injury to anticipating vulnerability. However, as demonstrated in the current evidence synthesis, technological ambition currently outpaces ecological validation in many of these predictive models.

Thus, the trajectory of the field can be interpreted not simply as technological advancement, but as a gradual reframing of fall prevention within a broader preventive aging paradigm.

### 4.2 Detection technologies: technical maturity and real-world constraints

Wearable IMU-based systems represent the most technically mature segment of the literature. Across multiple studies, sensitivity values in laboratory settings frequently exceed 85–90%, and algorithmic performance has steadily improved with machine learning integration (21). Compared with vision-based systems, wearable inertial sensors demonstrate greater robustness under variable lighting conditions and reduced infrastructure demands.

However, technical accuracy alone does not ensure public health effectiveness. Real-world deployment studies reveal several persistent challenges. False-positive alarms remain a recurrent issue, particularly during high-dynamic non-fall activities (29). Excessive alerts may erode user trust and reduce long-term adherence. In addition, wearable systems require consistent device placement, battery maintenance, and user compliance—factors that are particularly sensitive in frail or cognitively impaired populations (11).

In contrast, vision- and depth-based systems eliminate the need for wearable adherence and may offer advantages in institutional environments (18, 24). Yet these systems introduce concerns related to privacy, environmental sensitivity, and installation costs. Performance frequently declines in cluttered or multi-occupant settings. Thus, while non-wearable approaches reduce compliance burden, they shift complexity toward environmental infrastructure and ethical governance.

Collectively, detection technologies demonstrate strong technical validation but incomplete translational readiness. The primary limitation is no longer algorithmic feasibility, but ecological robustness and sustainable integration into daily life (30).

### 4.3 Pre-impact prediction: conceptual advancement, limited ecological validation

Pre-impact fall prediction systems represent a conceptual advancement beyond post-fall detection. By analyzing short temporal windows of kinematic instability, these systems aim to provide sub-second lead times that could theoretically enable protective interventions such as wearable airbags or assistive activation (16).

Experimental studies report lead times typically ranging between approximately 100 and 600 milliseconds (16). While promising, these results are predominantly derived from simulated fall protocols (21). Real-world falls among older adults often involve slower descent velocities, compensatory reactions, and complex environmental interactions not fully replicated in laboratory paradigms (8).

Compared with post-impact detection, predictive systems face a more delicate trade-off between sensitivity and false alarm minimization (29). Because predictive thresholds operate within normal movement variability, even small misclassifications may substantially increase alert burden. Consequently, translational readiness remains limited, and external validation in frail older populations is still sparse.

Thus, pre-impact systems currently represent a high-potential but early-stage domain within the technological maturity gradient.

### 4.4 Multimodal architectures and edge intelligence: precision versus complexity

Multimodal sensor fusion and edge-computing implementations aim to enhance robustness by integrating complementary signals and reducing latency. Compared with single-modality IMU systems, multimodal architectures often demonstrate improved discrimination between falls and high-dynamic activities. On-device inference may also mitigate privacy concerns associated with cloud transmission.

However, increased hardware and algorithmic complexity introduces new barriers. Calibration demands, interoperability challenges, maintenance requirements, and elevated costs may constrain scalability—particularly in resource-limited settings (21). Few studies provide cost-effectiveness analyses or long-term deployment data (11).

This reveals a recurring tension in the field: incremental improvements in classification precision frequently come at the cost of system complexity. From a public health perspective, optimal solutions may not necessarily be those with maximal algorithmic sophistication, but those achieving acceptable accuracy within sustainable operational frameworks (31).

### 4.5 Implementation readiness and health system alignment

Across technological categories, a consistent gap emerges between laboratory validation and implementation readiness. The majority of studies rely on controlled experimental datasets, while longitudinal community-based evaluations remain scarce.

For fall detection systems to produce meaningful population-level impact, they must align with care workflows, emergency response pathways, and caregiver communication structures. High sensitivity without operational integration may increase workload without improving outcomes. Conversely, overly conservative thresholds risk missing clinically relevant events.

Implementation science perspectives emphasize feasibility, adoption, sustainability, and system integration (32, 33). Few studies report adherence trajectories, caregiver response times, or long-term user acceptance. Without these data, scalability remains uncertain despite promising technical metrics.

Therefore, future research should expand beyond algorithm optimization toward structured implementation evaluation.

### 4.6 Ethical governance, equity, and the digital divide

Technological progress must also be examined through ethical and equity lenses. Advanced multimodal systems may disproportionately benefit regions with established digital infrastructure and financial resources (34). Populations with limited digital literacy or reduced economic access may face barriers to adoption.

Vision-based systems raise privacy concerns, particularly in private residential environments. Even wearable devices may generate concerns related to continuous monitoring and autonomy (35). Transparent data governance frameworks and user-centered design approaches are essential to maintain trust (36).

Emerging distributed learning architectures and edge computing strategies offer potential mitigation of privacy risks, yet regulatory harmonization and standardization remain underdeveloped (37). Ethical-by-design principles must accompany technical innovation to ensure that fall prevention technologies enhance, rather than compromise, dignity and autonomy.

### 4.7 Toward a predictive–preventive–personalized continuum

Rather than representing discrete categories, detection, prediction, and personalization can be conceptualized as a continuum of functional capability (38). Detection systems document injury events after impact; predictive systems attempt to identify instability patterns during movement; and personalized approaches seek to dynamically adapt thresholds and responses according to individual biomechanical profiles and contextual characteristics.

Current evidence indicates that detection technologies are technically mature yet operationally incomplete in real-world settings. Predictive systems demonstrate conceptual advancement but remain ecologically under-validated, particularly among frail and heterogeneous older populations. Personalized and ecosystem-level models, including digital twin–inspired frameworks and adaptive learning architectures, remain largely exploratory (39).

Advancement along this continuum requires staged methodological and translational maturation. Specifically, progress should involve robust external validation across diverse populations, longitudinal evaluation of adherence and usability, integration into clinical and caregiving workflows, formal health economic assessment, and alignment with regulatory and policy frameworks (40). Without such sequential progression, technological innovation risks remaining confined to experimental environments (32).

Only through systematic maturation across these domains can fall-related technologies transition from promising prototypes to scalable public health tools capable of supporting safe, independent, and dignified aging.

## 5 Conclusion

This scoping review provides a structured mapping of contemporary fall detection and pre-impact prediction technologies within aging and public health contexts. Over the past two decades, substantial technological progress has been achieved, particularly in wearable inertial measurement systems demonstrating strong performance in controlled laboratory environments. Nevertheless, translational maturity remains uneven across modalities.

Wearable IMU-based post-fall detection systems currently exhibit the highest level of technical validation, yet evidence regarding long-term adherence, workflow integration, and real-world sustainability remains limited. Pre-impact prediction approaches represent a meaningful conceptual shift toward anticipatory risk management; however, their ecological validity in frail and heterogeneous older populations requires robust external validation. Vision-based and multimodal architectures provide complementary advantages but introduce challenges related to privacy, environmental sensitivity, system complexity, and scalability.

Across technological streams, a consistent gap persists between algorithmic performance and population-level implementation. Technical accuracy alone is insufficient to ensure public health impact. Sustainable deployment depends on usability, integration into caregiving systems, cost-effectiveness, and ethical governance.

Rather than discrete technological phases, current evidence supports a functional continuum progressing from reactive detection toward predictive and personalized prevention. Advancing along this continuum will require ecologically valid datasets, multicenter real-world validation, longitudinal adherence assessment, implementation science frameworks, and economic evaluation.

Ultimately, intelligent fall-prevention technologies hold considerable promise for enabling safe, independent, and dignified aging. Their successful transition from experimental prototypes to scalable public health tools will depend on interdisciplinary collaboration, evidence-informed deployment strategies, and governance structures that prioritize equity, autonomy, and long-term sustainability.
